# Level of Evidence for Reliability, Validity, and Responsiveness of Physical Capacity Tasks Designed to Assess Functioning in Patients With Low Back Pain: A Systematic Review Using the COSMIN Standards

**DOI:** 10.1093/ptj/pzy159

**Published:** 2018-12-18

**Authors:** Max Jakobsson, Annelie Gutke, Lidwine B Mokkink, Rob Smeets, Mari Lundberg

**Affiliations:** 1 Back in Motion Research Group, Department of Orthopaedics, Institute of Clinical Sciences, University of Gothenburg, Mölndal Hospital, Göteborgsvägen 31, 431 80 Mölndal, Gothenburg, 41326 Sweden; 2 Division of Physiotherapy, Department of Health and Rehabilitation, Institute of Neuroscience and Physiology, University of Gothenburg; 3 Department of Epidemiology and Biostatistics, Amsterdam Public Health Research Institute, VU University Medical Center, Amsterdam, the Netherlands; 4 Department of Rehabilitation Medicine, Research School of CAPHRI, Maastricht University, Maastricht, the Netherlands; and CIR Revalidatie, Eindhoven, the Netherlands; 5 Division of Physiotherapy, Department of Health and Rehabilitation, Institute of Neuroscience and Physiology, University of Gothenburg; Department of Orthopaedics, Sahlgrenska University Hospital, Gothenburg, Sweden; and Division of Physiotherapy, Department of Neurobiology, Care Sciences and Society (NVS), Karolinska Institutet, Stockholm, Sweden

## Abstract

**Background:**

Physical capacity tasks (ie, observer-administered outcome measures that comprise a standardized activity) are useful for assessing functioning in patients with low back pain.

**Purpose:**

The purpose of this study was to systematically review the level of evidence for the reliability, validity, and responsiveness of physical capacity tasks.

**Data Sources:**

MEDLINE, CINAHL, PsycINFO, Scopus, the Cochrane Library, and relevant reference lists were used as data sources.

**Study Selection:**

Two authors independently selected articles addressing the reliability, validity, and responsiveness of physical capacity tasks, and a third author resolved discrepancies.

**Data Extraction and Quality Assessment:**

One author performed data extraction, and a second author independently checked the data extraction for accuracy. Two authors independently assessed the methodological quality with the Consensus-Based Standards for the Selection of Health Measurement Instruments (COSMIN) 4-point checklist, and a third author resolved discrepancies.

**Data Synthesis and Analysis:**

Data synthesis was performed by all authors to determine the level of evidence per measurement property per physical capacity task. The 5-repetition sit-to-stand, 5-minute walk, 50-ft (∼15.3-m) walk, Progressive Isoinertial Lifting Evaluation, and Timed “Up & Go” tasks displayed moderate to strong evidence for positive ratings of both reliability and construct validity. The 1-minute stair-climbing, 5-repetition sit-to-stand, shuttle walking, and Timed “Up & Go” tasks showed limited evidence for positive ratings of responsiveness.

**Limitations:**

The COSMIN 4-point checklist was originally developed for patient-reported outcome measures and not physical capacity tasks.

**Conclusions:**

The 5-repetition sit-to-stand, 50-ft walk, 5-minute walk, Progressive Isoinertial Lifting Evaluation, Timed “Up & Go,” and 1-minute stair-climbing tasks are promising tests for the measurement of functioning in patients with chronic low back pain. However, more research on the measurement error and responsiveness of these tasks is needed to be able to fully recommend them as outcome measures in research and clinical practice.

Research suggests that low back pain (LBP) causes more years lived with disability than any other condition.^1^ Many patients with acute LBP recover quickly, but the pain becomes a chronic condition for some, with significant consequences related to restricted functioning.^2^ The measurement of functioning is complex and covers multidimensional constructs.^3^ Functioning is divided into 3 domains according to the International Classification of Functioning, Disability, and Health (ICF). The body functions and structures domain refers to psychological and physiological processes and anatomical structures; the activity domain refers to people's ability to perform activities in their daily life; and the participation domain describes people's interactions with their sociocultural environment (eg, when buying groceries, participating in sports, or working).^3^ Researchers have argued for the use of outcome measures that specifically measure functioning in the activity domain.^4–6^

The ICF activity domain is typically measured with patient-reported outcome measures (PROMs), with which patients rate their perceived ability to perform various activities in their usual environment.^7–11^ PROMs are relatively time saving, easy to administer, and, importantly, include the patient's perspective.^12^ However, PROMs as used in LBP research have shown both floor and ceiling effects as well as low- to very-low-quality evidence for content validity.^13,14^ Clinical experience and scientific evidence also indicate frequent discrepancies between how patients score PROMs and how they actually move and perform activities when observed in the clinic.^15–17^ Several authors^5,6,16–18^ have therefore recommended the use of physical capacity tasks that measure the capacity qualifier of the ICF activity domain,^5,6,16–18^ defined as “the ability to execute a task or an action in a standardized environment.”^3^ In a physical capacity task, a patient performs a standardized activity that is administered by an observer using predefined criteria such as repetition counts or task timing.^18^

Physical capacity tasks are designed to assess what patients actually can do rather than what they think they can do, and can therefore capture important information about functioning that PROMs often do not.^17,19,20^ Physical capacity tasks also appear to be less influenced by education level and language skills compared with PROMs.^17,18,21,22^

All outcome measures should have sufficient support for their reliability, validity, and responsiveness in order to be used in research or in clinical practice. Otherwise, there is a significant risk of imprecise or biased results that might lead to incorrect conclusions regarding the patient's level of functioning.^23–25^ However, to our knowledge, no study has yet summarized the level of evidence of measurement properties of physical capacity tasks for patients with LBP.

The objective of this study was to systematically review the level of evidence of the measurement properties of physical capacity tasks that are designed to assess functioning in patients with LBP.

## Methods

The study protocol was registered at the International Prospective Register of Systematic Reviews (PROSPERO; http://www.crd.york.ac.uk/PROSPERO; registration number: CRD42016042011). We followed the working procedure developed by the Consensus-Based Standards for the Selection of Health Measurement Instruments (COSMIN) for conducting a systematic review of measurement properties.^23^ The systematic review was reported according to the Preferred Reporting Items for Systematic Reviews and Meta-Analyses (PRISMA).^26^

### Data Sources and Searches

The electronic data sources were MEDLINE (through the Ovid interface), CINAHL (EBSCOhost), PsycINFO (Ovid), Scopus (Elsevier), and the Cochrane Library (Wiley). The reference lists of the articles from the electronic data sources that were identified for full-text reading were used as additional data sources.

A search strategy was developed in collaboration with 2 medical librarians who also performed the search (eAppendix 1, available at https://academic.oup.com/ptj). The search strategy included 4 main filters, specifically tailored for each electronic information source: target population (ie, LBP), the construct of interest (ie, capacity), type of outcome measures (ie, physical capacity tasks), and article type (ie, articles on the reliability, validity, and responsiveness of outcome measures). The search strategy was based on a preliminary search strategy as described in the PROSPERO protocol. Improvements of the preliminary search strategy included: (1) adaptation of a validated search filter for measurement properties;^27^ (2) addition of the specific names of all physical capacity tasks identified by the preliminary search strategy; (3) use of the Ovid interface rather than PubMed for MEDLINE due to the benefit of positional operators; and (4) the addition of supplementary search terms for LBP. The first search with the final search strategy was performed on May 11, 2017 (no limitations for publication period) and an updated search was performed on August 29, 2018 (publication period from January 1, 2016). No restrictions were applied for language. Two authors (M.J. and either A.G. or M.L.) independently performed a hand search of the reference lists of all articles that had been identified for full-text reading.

### Study Selection

Two authors (M.J. and either A.G. or M.L.) independently screened the titles and abstracts of articles from the electronic and hand searches. A third author (R.S.) was consulted to resolve the disagreement if consensus could not be reached on the eligibility of articles for full-text review. Two authors (M.J. and A.G., R.S., or M.L.) then independently reviewed the full-text articles for eligibility, and a third author (A.G., R.S., or M.L.) was consulted if needed for consensus.

Articles that met 4 criteria (target population, construct, outcome measure, and article type) were included.

#### Target population

Patients in the study population were at least 18 years old and had had LBP for 6 weeks or more.^28^ Articles that contained pregnant participants or participants with fibromyalgia, confirmed rheumatic diseases, infections, tumors, osteoporosis, fractures, structural deformities (eg, scoliosis), or cauda equina syndrome were excluded unless data were presented specifically for patients who met the eligibility criteria.

#### Construct

The test was a measure of “capacity” under the ICF activity domain, defined as the ability to execute a task or an action in a standardized environment.^3^

#### Outcome measure

The test was a physical capacity task, defined as a standardized test that is used for an evaluative purpose and that is administered by an observer, includes an activity (as classified by the ICF) that is performed in a standardized setting, and requires readily available, low-cost, and portable equipment. Articles that exclusively investigated test batteries and did not present results for individual physical capacity tasks were excluded. If an article cited an original test manual that could then not be obtained, the test was excluded.

#### Article type

The article presented original data reporting the reliability (including reliability, measurement error, and internal consistency), validity (including content validity, construct validity, and criterion validity), or responsiveness of physical capacity tasks.^29^

### Data Extraction and Quality Assessment

A pilot version of a data extraction form was developed by the first author (M.J.) and piloted by 3 authors (M.J., R.S., and M.L.) on 5 randomly selected, included articles. The data extraction form was modified in response to the pilot to include the following 6 data items: (1) patient sample characteristics; (2) eligibility criteria; (3) setting; (4) procedure and equipment for performing the physical capacity tasks; (5) results of the measurement properties, and (6) minimal important change. Minimal important change is not considered a measurement property in the COSMIN taxonomy^29^ but was included in the data extraction form because this measure is necessary to determine the level of evidence for measurement error.^30^ Data from all included articles were subsequently extracted by 1 author (M.J.) and then independently checked for accuracy by a second author (A.G., R.S., or M.L.).

Two authors (M.J. and M.L.) independently assessed all of the included studies for methodological quality using the COSMIN 4-point checklist,^31,32^ and a third author (A.G., R.S., or L.B.M.) was consulted if needed to reach consensus. The COSMIN 4-point checklist is specifically designed to determine methodological quality scores of studies of measurement properties. The scores (excellent, good, fair, or poor) are assigned for each measurement property using the “worst score counts method.”^31,32^ The item for sample size in the checklist was excluded from the “worst score counts method” as recommended when performing systematic reviews of measurement properties of physical capacity tasks.^33^ The rating authors (M.J. and M.L.) pretested and then discussed the ratings of the checklist with a third author (L.B.M.) to achieve consistency in scoring, as recommended to ensure the validity of the scoring method.^34^

### Data Synthesis and Analysis

A “best-evidence synthesis” approach was performed by consensus of all authors to synthesize the level of evidence of measurement properties per physical capacity task.^35^ This procedure is similar to the Grading of Recommendations Assessment, Development, and Evaluation (GRADE),^36^ but different in that the best-evidence synthesis determines the level of evidence for the results of measurement properties, rather than the results of clinical trials.^23^ First, the results of each measurement property for each physical capacity task were rated as “positive,” “negative,” or “indeterminate” according to result rating criteria accepted with consensus in an international Delphi study (Tab. 1).^30^ Then, the level of evidence for the ratings of measurement properties was determined on the basis of the consistency of the result ratings of the measurement properties, the sample size of the combined eligible articles, and the methodological quality of the articles. Multiple articles were combined if they concerned the same physical capacity task and included samples with comparable characteristics. The possible levels of evidence were assigned according to 5 criteria (strong, moderate, limited, unknown, and conflicting) used in previous systematic reviews of the reliability, validity, and responsiveness of physical capacity tasks.^33,35^

**Table 1.** Criteria for Result Ratings of Measurement Properties*^a^*

Measurement Property	Rating*^b^*	Criterion for Result Rating
Reliability	+	ICC or weighted κ ≥0.70
	?	ICC or weighted κ not reported
	−	Criteria for “+” not met
Measurement error	+	SDC or LoA < MIC*^c^*
	?	MIC not defined
	−	Criteria for “+” not met
Hypothesis testing for construct validity	+	75% of the results in accordance with the hypotheses
	?	No hypotheses defined
	−	Criteria for “+” not met
Criterion validity	+	Correlation with gold standard ≥ 0.70 or AUC ≥ 0.70
	?	Not all information for “+” reported
	−	Criteria for “+” not met
Responsiveness	+	75% of the results in accordance with the hypotheses or AUC ≥ 0.70
	?	No hypotheses defined
	−	Criteria for “+” not met

^*a*^Based on Prinsen et al.^30^ AUC = area under the receiver operating characteristic curve; ICC = intraclass correlation coefficient; LoA = limits of agreement; MIC = minimal important change; SDC = smallest detectable change.

^*b*^+ = positive rating; ? = indeterminate rating; − = negative rating.

^*c*^This evidence can come from different studies.

#### Strong

This criterion encompassed consistent result ratings in at least 2 good-quality articles or at least 1 excellent-quality article, with a total sample size of eligible articles equal to or greater than 100.

#### Moderate

This criterion encompassed consistent result ratings in at least 2 fair-quality articles or 1 good-quality article, with a total sample size of eligible articles equal to or greater than 50.

#### Limited

This criterion encompassed at least 1 fair-, good-, or excellent-quality article, with a total sample size of eligible articles of 25 to 49.

#### Unknown

This criterion encompassed the following 4 options: indeterminate result ratings, all eligible articles were of poor methodological quality, the total sample size of eligible articles was less than 25, or conflicting result ratings.

#### Conflicting

This criterion encompassed conflicting findings.

If an article lacked a priori hypotheses for validity and responsiveness, we extracted what the authors had expected in relation to those measurement properties from the description found in the article. We generated our own hypotheses for validity and responsiveness based on these descriptions, which were then added to the data synthesis (eAppendixes 2 and 3, available at https://academic.oup.com/ptj).

### Role of the Funding Source

This review was supported by AFA Försäkring 120216, the Eurospine Research Grants 8-2014, the Health and Medical Care Executive Board of the Västra Götaland Region, and Vetenskapsrådet 2015-02511. The funders played no role in the study design, data analysis, interpretation of data, or writing of the manuscript. The views expressed here are those of the authors and do not necessarily reflect those of the funding sources.

## Results

### Study Selection and Characteristics

The search of electronic data sources and the hand search of reference lists resulted in 7900 articles after duplicates had been removed (Figure). Of these, 25 articles fulfilled the eligibility criteria (Tab. 2). The included articles comprised 18 physical capacity tasks that involved the following activities: walking (10 tasks), stair-climbing (1 task), lifting (3 tasks), rising from a chair (2 tasks), a combination of walking/rising from a chair (1 task), and a combination of walking and carrying (1 task) (Tab. 3).

**Figure.**
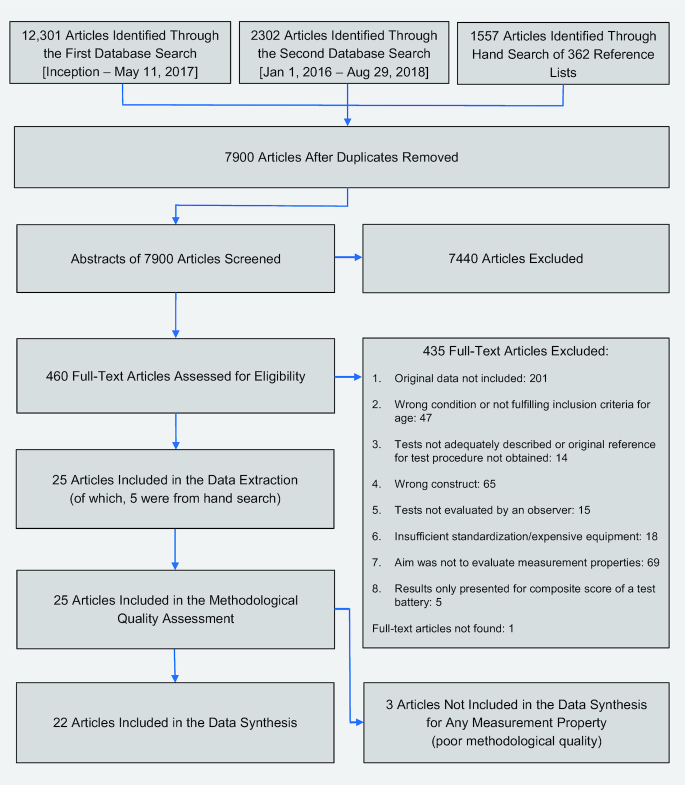
Flow of information through the different phases of the systematic review.

**Table 2.** Characteristics of Included Studies in Alphabetical Order of First Authors’ Names*^a^*

Study	Criteria for Patient Inclusion	Sample Size (% Women)	Age, Mean (SD) [Range]	Setting (Country)	Back Pain Duration*^b^*	Back Pain Intensity*^b^*	Patient-Reported Disability, Mean (SD)	Eligible Physical Capacity Task
Andersson et al^51^ (2010)	Nonspecific LBP for >3 mo	198 (47)	41.9 (9.1)	Tertiary care (the Netherlands)	Median: 24.0 mo (IQR: 12.0–73.0 mo)	VAS: 48.9 (24.7)	RMDQ: 13.8 (3.6)	1 min of stair climbing, 5-min walk, 50-ft*^c^* walk, PILE, sit-to-stand
Armstrong et al^52^ (2005)	LBP for ≥6 mo	10 (80)	40.8 (11.3)	Tertiary care (United Kingdom)	77.1 (35.8) mo	VAS: 46 (23)		Shuttle walking test
Campbell et al^38^ (2006)	LBP for >12 mo, considered for surgical stabilization of the spine (unspecified LBP, spondylolisthesis, or postlaminectomy)	250 (44)	40 (8.7) [19–55]	Hospitals (United Kingdom)	7.6 (6.7) y		ODI for improved: 42.6 (13.6); ODI for stable: 46.8 (13.7); ODI for deteriorated: 51.8 (13.5)	Shuttle walking test
Conway et al^20^ (2011)	Lumbar spinal stenosis	12 (25)	66.3 (9.8) [53–81]	Tertiary care (United States)	4.7 (1.5) y	VAS: 39.9 (27.0)	ODI: 48.9 (11.6); QBPDS: 48.2 (16.8)	Self-paced walking test
Deen et al^39^ (2000)	Lumbar spinal stenosis	28 (39.3)	73.8 [57–91]					Treadmill examination (only the test parameter “total ambulation”)
Gautschi et al^15^ (2016)	Lumbar disk herniation, lumbar spinal stenosis, and lumbar degenerative disk disease scheduled for lumbar spine surgery	253 (42.3)	58.4 (16.1)	Hospitals (Switzerland)		VAS: 39.1 (28.5); VAS for the leg: 48.8 (29.0)	RMDQ: 11.8 (5.2); ODI: 48.4 (20.8)	Timed “Up & Go” Test
Gautschi et al^45^ (2016)	Lumbar disk herniation, lumbar spinal stenosis, and lumbar degenerative disk disease scheduled for lumbar spine surgery	136 (44.1)	57.7 (15.8)	Hospitals (Switzerland)		VAS: 45.6 (17.6); VAS for the leg: 5.5 (2.6)	ODI: 45.6 (17.6); RMDQ: 11.3 (5.1)	Timed “Up & Go” Test
Gautschi et al^44^ (2017)	Lumbar disk herniation, lumbar spinal stenosis, and lumbar degenerative disk disease scheduled for lumbar spine surgery	100 (43)	56.2 (16.1)	Hospitals (Switzerland)		VAS: 38.0 (10); VAS for the leg: 55.0 (28)	RMDQ: 12 (5); ODI: 46 (18)	Timed “Up & Go” Test
Kahraman et al^47^ (2016)	Nonspecific LBP for >12 wk	38 (37)	35 (10)		7 (3) mo	VAS with rest: 21.3 (15.1); VAS with activity: 64.5 (20.1)	ODI: 22.6 (14.2)	30-s chair stand test
Lee et al^19^ (2001)	LBP with mechanical, structural, or nonspecific origins	83 (57.8)	45.64 (10.12) [24–65]	Orthopedic spine clinic (United States)	108.2 (127.9) mo	VAS: 36.9 (27.0)	RMDQ: 10.4 (6.1)	5-min walk, 50-ft walk, 5-repetition sit-to-stand
Magnussen et al^37^ (2004)	LBP for >8 wk	32 (66%)	38.1 (9.2)	Tertiary care (Norway)			RMDQ: 10.0 (4.0); FFbH-R: 22.5 (4.9)	Lift test
Ocarino et al^53^ (2009)	Nonspecific LBP for ≥3 mo	30	43.16 (11.23)	University clinic (Brazil)	42.3 (80.6) mo		RMDQ: 9.9 (3.7)	50-ft walk, sit-to-stand
Odebiyi et al^54^ (2007)	LBP for ≥3 mo with radiating leg pain	23	[25–65]	Two hospitals (Nigeria)		VAS for men: 45.2 (19.4); VAS for women: 64.1 (22.4)	RMDQ for men: 8.4 (5.3); RMDQ for women: 9.6 (4.9)	50-ft walk, 5-min walk, 5-repetition sit-to-stand
Pratt et al^40^ (2002)	Lumbar spinal stenosis	29 (41)	69 [49–82]	Orthopedic hospital (United Kingdom)			ODI: 39.8 (16.5)	Shuttle walking test
Rainville et al^41^ (2012)	Lumbar spinal stenosis	50 (42)	68 (7.9) [48–86]	Spine center of a hospital (United States)		VAS: 61; VAS for the leg: 54	ODI: 35	Motorized treadmill test, self-paced walking test
Simmonds et al^17^ (1998)	Nonspecific, mechanical LBP	44 (63.6)	42.6	Orthopedic clinic (United States)	12.4 (20.8) [1–72] mo	VAS: 27.3 (23.9) [0.0–7.2]	RMDQ: 8.3 (6.0)	1 min of stair climbing, 5-min walk, 50-ft walk, 50-ft walk (preferred speed), 5-repetition sit-to-stand
Smeets et al^5^ (2006)	Nonspecific LBP for >3 mo	53 (52.8)	43.19 (9.27)	Tertiary care (the Netherlands)	53.4 (67.7) mo	VAS: 44.5 (23.5)	RMDQ: 13.2 (4.2)	1-min of stair climbing, 5-min walk, 50-ft walk, PILE, 5-repetition sit-to-stand
Soer et al^55^ (2006)	LBP for >3 mo	53 (39.6)	38.5 (9.8)		250 (375) wk		RMDQ: 9.2 (5.5)	PILE
Staartjes and Schroder^46^ (2018)	Lumbar disk herniation, lumbar spinal stenosis, spondylolisthesis, degenerative disk disease	157 (49)	49.9 (14.1)	Outpatient spine surgery clinic (the Netherlands)		VAS: 5.8 (2.8); VAS for the leg: 7.4 (2.0)	ODI: 43.0 (17.6); RMDQ: 11.7 (5.3)	5-repetition sit-to-stand
Strand et al^56^ (2002)	LBP for >3 mo	114 (60)	43.9 (10.6)	Tertiary care (Norway)	10 (9.0) y	VAS: 52.6 (20.5)	Disability Rating Index: 56.2 (13.3)	Lift test
Strand et al^48^ (2011)	LBP for >3 mo	98 (49.0)	37.4 (10.4) [18–60]	Tertiary care (Norway)			RMDQ: 11.8 (4.4); FFbH-R: 7.9 (5.3)	15-m walk test, lift test (modified), PILE
Taylor et al^49^ (2001)	Mechanical LBP with or without sciatica for ≥6 mo	44 (59.2)	48.2 (9.1)	Tertiary care (Great Britain)			ODI: 28.5	
Teixeira da Cunha-Filho et al^50^ (2010)	LBP for >3 mo	30 (63.3)	33.0 (10.6)	Tertiary care (Brazil)		VAS: 44 (26)	RMDQ: 9.8 (5.8)	50-ft walk, 5-min walk, 5-repetition sit-to-stand, Timed “Up & Go” Test
Tomkins et al^42^ (2009)	Lumbar spinal stenosis	45 (57.8)	66.9 (9.6)		10 (11) y; leg pain: 7 (9) y			Self-paced walking test, treadmill protocol
Whitehurst et al^43^ (2001)	Lumbar spinal stenosis	57 (56.1)	Men: 69 (5); women: 70 (3)					Treadmill walking test, weight-carrying test

^*a*^FFbH-R = Hannover Functional Ability Questionnaire; IQR = interquartile range; LBP = low back pain; ODI = Oswestry Disability Index; PILE = Progressive Isoinertial Lifting Evaluation; QBPDS = Quebec Back Pain Disability Scale; RMDQ = Roland-Morris Disability Questionnaire; SD = standard deviation; VAS = 100-mm visual analog scale.

^*b*^Reported as mean (SD) [range] unless otherwise indicated.

^*c*^50 ft ≈ 15.3 m.

**Table 3.** Brief Descriptions of Included Physical Capacity Tasks

Physical Capacity Task	Activity	Quantification Measure	Equipment Needed
1-min stair climbing	Stair climbing	No. of stairs climbed in 1 min	A flight of stairs with handrails, stopwatch
30-s chair stand test	Rising from a chair	No. of repetitions (sitting to standing) performed in 30 s	Chair, stopwatch
5-repetition sit-to-stand	Rising from a chair	Seconds to complete 5 repetitions of sitting to standing	Chair, stopwatch
50-ft*^a^* walk	Walking	Seconds to complete a 50-ft course at maximum speed	Measuring tape, stopwatch, markers for indicating track end points
50-ft walk, preferred speed	Walking	Seconds to complete a 50-ft course at preferred speed	Measuring tape, stopwatch, markers for indicating track endpoints
5-min walk	Walking	Meters walked in 5 min	Measuring tape, stopwatch, markers for indicating track end points
Lift test	Lifting	Ordinal scale of no. of repetitions of lifting a box with a sandbag from floor to table and back	Table, 1.35-kg box with 5-kg sandbag
Lift test, modified	Lifting	No. of repetitions of lifting a box from floor to table and back in 1 min	Table, 1.35-kg box with 5-kg sandbag (4 kg for women)
Motorized treadmill test	Walking	Total walking time and distance walked at preferred speed (nonmodifiable during the test) at the moment when walking-related symptoms make the participant stop	Treadmill, stopwatch
Progressive isoinertial lifting evaluation	Lifting	Weight (in kg) of the box during the last completed cycle^48^/no. of completed lifting cycles^5,51^	Standardized box, an assortment of weights
Self-paced walking test	Walking	Total walking time, speed, and distance walked at the moment when walking-related symptoms make the participant stop	Distance instrument, stopwatch
Shuttle walking test	Walking	Meters walked until the participant fails to complete a predefined “shuttle” in the time allocated	Standardized audiotape, measuring tape, markers for indicating track end points
Timed “Up & Go” Test	Rising from a chair and walking	Seconds to complete rising from a chair, walking 3 m, turning around, walking back to the chair, and sitting down	Chair, stopwatch
Treadmill examination, 1.2 mph*^b^*	Walking	Total time walked at 1.2 mph on a treadmill at the moment when walking-related symptoms make the participant stop (time limit: 15 min)	Treadmill, stopwatch
Treadmill examination, preferred speed	Walking	Total time walked at preferred speed on a treadmill at the moment when walking-related symptoms make the participant stop (time limit: 15 min)	Treadmill, stopwatch
Treadmill protocol	Walking	Total distance, time, and average speed walked on a treadmill at preferred speed (modifiable during the test) at the moment when symptoms of lumbar spinal stenosis or other reasons make the participant stop (time limit: 30 min)	Treadmill, stopwatch
Treadmill walking test	Walking	Distance walked at 53.6 m/min on a treadmill at the moment when pain or fatigue makes the participant stop or when 70% of the predicted maximum heart rate (70[220 – age]/100) is reached	Treadmill, stopwatch, heart rate monitor
Weight-carrying test	Walking and carrying	Time needed to walk 20 m as quickly as possible while carrying dumbbells equaling 10% of the person's weight	Stopwatch, an assortment of dumbbells

^*a*^50 ft ≈ 15.3 m.

^*b*^1.2 miles ≈ 1.9 km.

One article included patients with a pain duration of at least 6 weeks (“subacute” LBP^28^),^37^ whereas the rest included patients with a pain duration of at least 12 weeks (“chronic” LBP^28^). Eleven articles included patients with back-related diagnoses known to affect walking capacity severely.^15,20,38–46^ Of these, 1 article included patients with lumbar spondylolisthesis and postlaminectomy,^38^ 6 articles included patients with lumbar spinal stenosis,^20,39–43^ 3 articles included patients with lumbar disk herniation and lumbar spinal stenosis,^15,44,45^ and 1 article included patients with lumbar disk herniation, lumbar spinal stenosis, and spondylolisthesis.^46^

The included articles investigated 5 measurement properties: reliability, measurement error, construct validity (hypothesis testing), criterion validity, and responsiveness.

### Quality Assessment and Data Synthesis

#### Reliability

The results for reliability are shown in Table 4. Regarding methodological quality, 11 articles investigated reliability.^5,17,37,39,40,42,46–50^ Three studies of reliability in 1 article^48^ were rated as poor for methodological quality and therefore excluded from the best-evidence synthesis. The poor scores were due to the fact that patients received treatment between the first and second administration of the tasks.

**Table 4.** COSMIN Methodological Quality Ratings, Result Ratings, and Level of Evidence for Reliability and Measurement Error as Well as Minimal Important Change Per Physical Capacity Task*^a^*

Physical Capacity Task	Study	Study Design*^b^*	Sample Size	Reliability			Measurement Error			MIC*^c^*
				COSMIN Score^32^	Result Ratings (+/?/−)^30^	Best-Evidence Synthesis: Level of Evidence	COSMIN Score^32^	Result Ratings (+/?/−)^30^	Best-Evidence Synthesis: Level of Evidence	
1-min stair climbing	Andersson et al^51^ (2010)	Test-retest	134			Test-retest: moderate (+)	Poor*^d^*	SDC: 28.6 steps*^e^* (−)	Test-retest: moderate (−)	14.5 steps
	Smeets et al^5^ (2006)	Test-retest	53	Good	ICC(1,1): 0.96 (+) (95% CI: 0.93–0.98)		Good	LoA: ±14.7 steps (−)		
30-s chair stand test	Kahraman et al^47^ (2016)	Intrarater	38	Good*^f^*	ICC(2,1): 0.94 (+) (95% CI: 0.89–0.97)	Intrarater: limited (+)			No information	
5-repetition sit-to-stand	Andersson et al^51^ (2010)	Test-retest	134			Test-retest: strong (+); interrater: unknown	Poor*^d^*	SDC: 11.6 s*^e^* (−)	Test-retest: strong (−); interrater: unknown	−4.1 s
	Simmonds et al^17^ (1998)	Test-retest (participants tested on different days)	44	Good*^f^*	ICC(1,k): 0.89 (+)		Good*^f^*	SDC: 6.38 s*^e^* (−)		
	Simmonds et al^17^ (1998)	Test-retest (participants tested on the same day)	44	Fair	ICC(1,1): 0.45 (−)		Fair	SDC: 18.46 s*^e^* (−)		
	Simmonds et al^17^ (1998)	Interrater	22	Fair	ICC(1,1): 0.99 (+)			SDC: 1.14 s*^e^* (+)		
	Smeets et al^5^ (2006)	Test-retest	53	Good	ICC(1,1): 0.91(+) (95% CI: 0.84–0.94)		Good	LoA: ±/7.6 s (−)		
	Staartjes and Schroder^46^ (2018)	Test-retest	66	Fair	0.97 (95% CI: 0.94–0.98)			SDC: 4.1 s*^e^*		
	Teixeira da Cunha-Filho et al^50^ (2010)	Test-retest	30	Fair	ICC(2,1): 0.99 (+)		Fair			
50-ft*^b,g^* walk	Andersson et al^51^ (2010)	Test-retest	133			Test-retest: strong (+); interrater: unknown	Poor*^d^*	SDC: 3.0 s*^e^* (−)	Test-retest: strong (−); interrater: unknown	−0.7 s
	Simmonds et al^17^ (1998)	Test-retest (participants tested on different days)	44	Good*^f^*	ICC(1,1): 0.80 (+)		Good*^f^*	SDC: 4.82 s*^e^* (−)		
	Simmonds et al^17^ (1998)	Test-retest (participants tested on the same day)	44	Fair	ICC(1,1): 0.99 (+)		Fair	SDC: 1.19 s*^e^* (−)		
	Simmonds et al^17^ (1998)	Interrater	22	Fair*^f^*	ICC(1,1): 0.99 (+)		Fair*^f^*	SDC: 0.61 s (+)		
	Smeets et al^5^ (2006)	Test-retest	52	Good	ICC(1,1): 0.76 (+) (95% CI: 0.61–0.85)		Good	LoA: ±3.9 s (−)		
	Strand et al^48^ (2011)	Test-retest	9	Poor*^d^*	ICC(2,1): 0.77 (+) (95% CI: 0.24–0.94)		Poor*^d^*	SDC: 0.6 s (+)		
	Teixeira da Cunha-Filho et al^50^ (2010)	Test-retest	30	Fair	ICC(2,1): 0.96 (+)					
50-ft walk^*g*^, preferred walking speed	Simmonds et al^17^ (1998)	Test-retest (participants tested on different days)	44	Good	ICC(1,1): 0.64 (−)	Test-retest: conflicting; interrater: unknown	Good*^f^*	SDC: 7.10 s*^e^* (?)	Unknown	
	Simmonds et al^17^ (1998)	Test-retest (participants tested on the same day)	44	Fair	ICC(1,1): 0.95 (+)		Fair	SDC: 2.74 s*^e^* (?)		
	Simmonds et al^17^ (1998)	Interrater	22	Fair*^f^*	ICC(1,1): 0.98 (+)		Fair	SDC: 1.52 s*^e^* (?)		
5-min walk	Andersson et al^51^ (2010)	Test-retest	132			Test-retest: strong (+)	Poor*^d^*	SDC: 105.1 m*^e^* (−)	Test-retest: moderate (−)	21.4 m
	Simmonds et al^17^ (1998)	Test-retest (participants tested on different days)	44	Good*^f^*	ICC(1,1): 0.99 (+)		Good*^f^*	SDC: 100.3 m*^e^* (−)		
	Smeets et al^5^ (2006)	Test-retest	53	Good	ICC(1,1): 0.89 (+) (95% CI: 0.81–0.93)		Good	LoA: ±82.7 m (?)		
	Teixeira da Cunha-Filho et al^50^ (2010)	Test-retest	30	Fair	ICC(2,1): 0.99 (+)					
Lift test	Magnussen^37^ (2004)	Interrater	32	Good*^f^*	κ: 1.00 (+) (95% CI: 1.00–1.00)	Interrater, subacute LBP: limited (+); test-retest, subacute LBP: limited (−)			No information	
	Magnussen^37^ (2004)	Test-retest	28	Good*^f^*	κ: 0.55 (−) (95% CI: 0.51–0.59)					
Lift test, modified	Strand et al^48^ (2011)	Test-retest	9	Poor*^d^*	ICC(2,1): 0.87 (+) (95% CI: 0.50–0.97)	Test-retest: unknown	Poor*^d^*	SDC: 6.5 lifts/min (−)	Unknown	2.5 lifts/min
Progressive isoinertial lifting evaluation	Andersson et al^51^ (2010)	Test-retest	126			Test-retest: moderate (+)	Poor*^d^*	SDC: 4.2 stages*^e^* (−)	Test-retest: moderate (−)	1.5 stages
	Smeets et al^5^ (2006)	Test-retest	50	Good	ICC(1,1)_:_ 0.92 (+) (95% CI: 0.87–0.96)		Good	LoA: ±2 stages (?)		
	Strand et al^48^ (2011)	Test-retest	9	Poor*^d^*	ICC(2,1): 0.91 (+) (95% CI: 0.65–0.98)		Poor*^d^*	SDC: 8.4 kg (?)		
Self-paced walking test	Tomkins et al^42^ (2009)	Test-retest	33	Fair	ICC(3,1): 0.98 (+)	Test-retest, LSS: limited (+)			No information	
Shuttle walking test	Armstrong et al^52^ (2005)	Test-retest				Test-retest: limited (+); test-retest, LSS: limited (+)	Good*^f^*	LoA: ±22 m (?)	Unknown	
	Pratt et al^40^ (2002)	Test-retest	29	Fair*^f^*	ICC(?,?): 0.92 (+)					
	Taylor et al^49^ (2001)	Test-retest	44	Fair*^f^*	ICC(1,1): 0.99 (+)		Fair	LoA: ±49.5 m (?)		
Timed “Up & Go” Test	Gautschi et al^44^ (2017)	Test-retest	18–62			Test-retest: moderate (+); interrater: unknown; test-retest, LDH/LSS/DDD: unknown	Poor*^d^*	Average SDC: 4.9 s (?)	Test-retest: unknown; test-retest, LDH/LSS/DDD: unknown	LDH/LSS/DDD: 3.4 s
	Simmonds et al^17^ (1998)	Test-retest (participants tested on different days)	44	Good*^f^*	ICC(1,k): 0.98 (+)		Good*^f^*	SDC: 2.74 s*^e^* (?)		
	Simmonds et al^17^ (1998)	Test-retest (participants tested on the same day)	44	Fair	ICC(1,1): 0.98 (+)		Fair	SDC: 1.16 s*^e^* (?)		
	Simmonds et al^17^ (1998)	Interrater	22	Fair*^f^*	ICC(1,1): 0.99 (+)		Fair*^f^*	SDC: 0.80 s*^e^*		
	Teixeira da Cunha-Filho et al^50^ (2010)	Test-retest	30	Fair	ICC(2,1): 0.92 (+)					
Treadmill examination, 1.2 mi/h*^h^*	Deen et al^39^ (2000)	Test-retest	28	Fair*^f^*	CCC: 0.90 (+)	Test-retest, LSS: limited (+)			No information	
Treadmill examination, preferred speed	Deen et al^39^ (2000)	Test-retest	28	Fair*^f^*	CCC: 0.96 (+)	Test-retest, LSS: limited (+)			No information	

^*a*^CCC = concordance correlation coefficient (comparable to ICC^67^); COSMIN = Consensus-Based Standards for the Selection of Health Measurement Instruments; DDD = lumbar degenerative disk disease; ICC = intraclass correlation coefficient; LBP = low back pain; LDH = lumbar disk herniation; LoA = limits of agreement; LSS = lumbar spinal stenosis; MIC = minimal important change; SDC = smallest detectable change. + = positive rating: ICC or weighted κ ≥ 0.70 (reliability), or SDC or LoA < MIC (measurement error); ? = indeterminate rating: ICC or weighted κ not reported (reliability) or MIC not defined (measurement error); − = negative rating: criteria for “+” not met.

^*b*^Intrarater reliability = when presenting repeatedly the same observations to 1 observer; interrater reliability = when presenting the same observations to 2 or more observers; test-retest reliability = when presenting the same task to the same subjects 2 or more times.^68^

^*c*^The MIC (defined as “the smallest change in score that is perceived as important by patients”^23^) was added for reference because this value is needed to determine the result ratings for measurement error.^66^

^*d*^Not included in the data synthesis because of the poor methodological score.^23^

^*e*^The SDC (defined as “the smallest change that can be detected by the measurement instrument, beyond measurement error”^23^) was calculated^23^ by the authors of the present systematic review by multiplying the standard error of measurement presented in the original article by 1.96 × $\sqrt {2}$.

^*f*^The rating changed after removal of the sample size item from the “worst score counts” summary in the COSMIN 4-point checklist.

^*g*^50 ft ≈ 15.3 m.

^*h*^1.2 miles ≈ 1.9 km.

With regard to best-evidence synthesis, a strong level of evidence for positive ratings for test-retest reliability was found for 3 tasks (50-ft [∼15.3-m] walk, 5-minute walk, and 5-repetition sit-to-stand tasks). A moderate level of evidence for positive ratings for test-retest reliability was found for 4 tasks (1-minute stair-climbing, Progressive Isoinertial Lifting Evaluation, and Timed “Up & Go” tasks). Four tasks were specifically evaluated for patients with back-related diagnoses known to severely affect walking capacity (self-paced walking test, shuttle walking test, and treadmill examination at 1.2 mph [∼1.9 km/h] and at preferred speed), and all of these had limited evidence for positive ratings for test-retest reliability. Intrarater reliability was assessed for 1 task (30-second chair stand test), which had limited evidence for a positive rating. Most of the tasks investigated for interrater reliability displayed unknown evidence due to small sample sizes.

#### Measurement error

The results for measurement error are shown in Table 4.

Regarding methodological quality, 8 articles investigated measurement error.^5,17,44,46,48,49,51,52^ Nine studies of measurement error in 3 articles^44,48,51^ were rated as poor and therefore excluded from the best-evidence synthesis. The poor scores were due to the fact that patients in the articles received treatment between the first and second administration of the tasks.

With regard to best-evidence synthesis, a moderate level of evidence for negative ratings for measurement error was displayed by the 1-minute stair-climbing, 5-minute walk, and Progressive Isoinertial Lifting Evaluation tasks. A strong level of evidence for negative ratings was displayed by 50-ft walk and 5-repetition sit-to-stand tasks. The negative ratings were a consequence of failure to meet the criterion stating that the smallest detectable change must be smaller than the minimal important change.^30^ The level of evidence was unknown for 4 tasks (50-ft walk [preferred speed] task, modified lift test, shuttle walking test, and Timed “Up & Go” task), because the minimal important change was not investigated.

#### Construct validity (hypothesis testing)

The results for construct validity (hypothesis testing) are shown in eTable 1 (available at https://academic.oup.com/ptj).

With regard to methodological quality, 12 articles investigated construct validity.^17,19,20,41,43,44,47,48,50,53–55^ Five studies of construct validity in 2 articles^53,54^ were rated as poor and therefore excluded from the best-evidence synthesis. The poor scores were due to an absence of a priori validity hypotheses (such as the hypothesized correlation between 2 outcome measures required for adequate validity).

With regard to best-evidence synthesis, a moderate level of evidence for positive ratings of construct validity was found for 6 tasks (50-ft walk task, 5-minute walk task, modified lift test, Progressive Isoinertial Lifting Evaluation, 5-repetition sit-to-stand task, and Timed “Up & Go” task). Limited evidence for positive ratings for construct validity was found for 4 walking tasks investigated specifically for patients with back-related diagnoses known to severely affect walking capacity (Timed “Up & Go” task, motorized treadmill test, treadmill walking test, and weight-carrying test).

#### Criterion validity

The results for criterion validity are shown in eTable 1.

With regard to methodological quality, 1 article investigated criterion validity^42^ and was rated as good for methodological quality and was therefore included in the best-evidence synthesis.

As regards best-evidence synthesis, the treadmill protocol displayed limited evidence for a positive rating for criterion validity for patients with lumbar spinal stenosis.

#### Responsiveness

The results for responsiveness are shown in eTable 2.

Regarding methodological quality, 7 articles investigated responsiveness.^38,41,48,49,51,56,57^ All studies of responsiveness in the articles received either fair or good scores and were therefore included in the best-evidence synthesis.

With regard to best-evidence synthesis, a limited level of evidence for positive ratings for responsiveness was found for 3 tasks (1-minute stair-climbing task, shuttle walking test, and 5-repetition sit-to-stand task). A moderate level of evidence for negative ratings was found for 3 tasks (50-ft walk task, modified lift test, and Progressive Isoinertial Lifting Evaluation). A limited level of evidence for negative ratings was found for 4 tasks (5-minute walk task, lift test, motorized treadmill test, and self-paced walking test). A limited level of evidence for a positive rating for responsiveness was found for 1 task (Timed “Up & Go” task) that was specifically for patients with back-related diagnoses known to severely affect walking capacity.

#### Summary of the level of evidence

A summary of the level of evidence is shown in Table 5.

**Table 5.** Overview of the Level of Evidence Per Measurement Property Per Physical Capacity Task*^a^*

PhysicalCapacity Task	Test-Retest Reliability	Interrater Reliability	Intrarater Reliability	Measurement Error	Hypothesis Testing	Criterion Validity	Responsiveness
1-min stair climbing	Moderate (+)	0	0	Moderate (−)	0	0	Limited (+)
30-s chair stand	0	0	Limited (+)	0	Limited (+)	0	0
5-repetition sit-to-stand	Strong (+)	Unknown	0	Strong (−)	Moderate (+)	0	Limited (+)
50-ft*^b^* walk	Strong (+)	Unknown	0	Strong (−)	Moderate (+)	0	Moderate (−)
50-ft walk, preferred speed	Conflicting	Unknown	0	Unknown	Conflicting	0	0
5-min walk	Strong (+)	0	0	Moderate (−)	Moderate (+)	0	Limited (−)
Lift test	Limited (−)	Limited (+)	0	0	0	0	Limited (−)
Lift test, modified	Unknown	0	0	Unknown	Moderate (+)	0	Moderate (−)
Motorized treadmill test	0	0	0	0	*Limited (+)^c^*	0	*Limited (−)^c^*
Progressive isoinertial lifting evaluation	Moderate (+)	0	0	Moderate (−)	Moderate (+)	0	Moderate (−)
Self-paced walking test	*Limited* (+)*^c^*	0	0	0	*Conflicting^c^*	0	*Limited (−)^c^*
Shuttle walking test	Limited (+), *Limited (+)^c^*	0	0	Unknown	0	0	Limited (+), *Limited (+)^c^*
Timed “Up & Go” Test	Moderate (+)	Unknown	0	Unknown	Moderate (+), *Limited (+)^c^*	0	*Limited (+)^c^*
Treadmill examination, 1.2 mph*^d^*	*Limited (+)^c^*	0	0	0	0	0	0
Treadmill examination, preferred speed	*Limited (+)^c^*	0	0	0	0	0	0
Treadmill protocol	0	0	0	0	0	*Limited (+)^c^*	0
Treadmill walking test	0	0	0	0	*Limited (+)*^c^**	0	0
Weight-carrying test	0	0	0	0	*Limited (+)^c^*	0	0

^*a*^+ = positive result rating; − = negative result rating; 0 = no information

^*b*^50 ft ≈ 15.3 m.

^*c*^The level of evidence marked in italics primarily concerns patients with back-related diagnoses known to severely affect walking capacity (eg, lumbar spinal stenosis, spondylolisthesis).

^*d*^1.2 miles ≈ 1.9 km.

The 5-repetition sit-to-stand task was the only physical capacity task that displayed positive ratings for more than 2 measurement properties: test-retest reliability (strong evidence), construct validity (moderate evidence), and responsiveness (limited evidence). The 50-ft walk, 5-minute walk, Progressive Isoinertial Lifting Evaluation, and Timed “Up & Go” tasks displayed moderate to strong evidence for positive ratings for both test-retest reliability and construct validity. The above-mentioned tasks, however, also displayed moderate to strong evidence for negative ratings for measurement error. The 1-minute stair-climbing task and shuttle walking test displayed positive ratings for responsiveness (limited evidence) as well as for test-retest reliability (moderate evidence for 1-minute stair-climbing task and limited evidence for shuttle walking test).

Of the walking tasks that were specifically investigated for patients with diagnoses known to severely affect walking capacity, the Timed “Up & Go” task and shuttle walking test were the only tasks that displayed positive ratings for more than 1 measurement property.

## Discussion

To our knowledge, this is the first study to determine the level of evidence for the reliability, validity, and responsiveness of physical capacity tasks designed to assess functioning for patients with LBP. The 5-repetition sit-to-stand, 50-ft walk, 5-minute walk, Progressive Isoinertial Lifting Evaluation, and Timed “Up & Go” tasks displayed moderate to strong evidence for positive ratings for both test-retest reliability and construct validity. These results suggest that a clinician or researcher can be confident that the scores of these tasks have a high degree of reliability and are likely to measure the constructs they are designed to measure.^23^ A previous systematic review investigated the reliability of physical capacity tasks, but not the validity and responsiveness.^58^ That review reported similar results for reliability, despite a different method for determining the level of evidence.^58^

The 1-minute stair climbing task, 5-repetition sit-to-stand task, shuttle walking test, and Timed “Up & Go” displayed limited evidence for positive ratings for responsiveness. These results suggest that these tasks appear to have the ability to detect changes in functioning after a treatment, although the evidence is limited. In contrast, most included tasks that were investigated for responsiveness displayed limited to moderate evidence for negative ratings. An important aspect of the definition of responsiveness in the COSMIN taxonomy is the ability to detect change over time specifically in the construct to be measured.^29^ However, most of the included articles investigated responsiveness by comparison with global rating scales of constructs such as “LBP-associated disability,”^51^ “general health improvements,”^38^ and “return to work,”^56^ all constructs that are different from those found in the physical capacity tasks. In contrast, several authors have recommended using global rating scales of the same constructs as the outcome measures of interest.^23^ For instance, the responsiveness of a walking task could preferably be investigated by comparing the results with a global rating scale concerning walking ability.^23^ Future high-quality studies with a priori hypotheses that include global rating scales of capacity could show clearer evidence for the responsiveness of physical capacity tasks.

A moderate to strong level of evidence for negative ratings for measurement error was displayed by 5 tasks because the smallest detectable change was larger than the minimal important change. Similar findings have been reported previously for PROMs of self-efficacy and health-related quality of life.^59,60^ The smallest detectable change (defined as “the smallest change that can be detected by the measurement instrument, beyond measurement error”^23^) should be smaller than the minimal important change (defined as “the smallest change in score that is perceived as important by patients”^23^) in order to detect changes that are as small as the minimal important change.^61^ The data of the systematic review suggest that the smallest detectable change of the investigated tasks appears to be too large to distinguish minimal important change from measurement error. Nevertheless, all negative ratings of measurement error were due to comparisons with minimal important change values determined for 5 tasks in a single article.^51^ That article investigated minimal important change values with a global rating scale of “LBP-related disability” rather than the constructs that the physical capacity tasks measure. In contrast, minimal important change is recommended to be compared with similar constructs as those in the outcome measures of interest.^61^ Moreover, the COSMIN 4-point scale is also not designed to assess the quality of studies that investigate minimal important change^32^ and therefore the methodological quality of the relevant article was undetermined.

We argue that the results of the systematic review are generalizable to most individuals with chronic LBP. Research suggests that a representative sample is required to generalize the results of measurement properties beyond the research setting.^23^ The patients in the systematic review comprise a heterogeneous sample, which is partly reflected in the differences in levels of pain and disability seen between the included studies (Tab. 2). Although we acknowledge that such heterogeneity can influence the results of physical capacity testing (eg, meters walked or weight lifted), there seems to be little evidence that the heterogeneity would affect the results of measurement properties. Not least, this is seen in the almost unanimous positive results for test-retest reliability and construct validity regardless of the characteristics of the study populations. A subset of the results is, however, primarily generalizable to patients with back-related diagnoses known to severely affect walking capacity such as lumbar spinal stenosis and spondylolisthesis (see the level of evidence marked in italics in Tab. 5).^62–64^ In the data synthesis of walking tasks, we therefore did not combine studies of such patients with other studies.

Predominantly positive ratings for single measurement properties were found for walking tasks specifically investigated for patients with back-related diagnoses known to affect walking capacity severely. However, only the Timed “Up & Go” task and shuttle walking test displayed positive ratings for more than 1 measurement property. This result appears to be due to a lack of studies investigating measurement properties with the same test protocol. For instance, 5 treadmill walking tasks with similar protocols^20,41–43^ were evaluated for patients with lumbar spinal stenosis, but these protocols were considered too heterogeneous for the results to be combined in the data synthesis. Physical capacity tasks investigated in future research might show a stronger level of evidence considering the promising results reported in these articles.

### Strengths and Limitations

A strength of the present review was the use of the COSMIN 4-point checklist for assessing the methodological quality of the included articles.^32^ To our knowledge, the checklist is the most widely used consensus-based quality assessment tool specifically developed for studies on measurement properties. During the course of this systematic review, the COSMIN checklist and the data synthesis procedure were updated.^65,66^ For instance, poor studies are included in the new data synthesis procedure.^65,66^ After weighing the pros and cons we decided to proceed with the original methodology as in our PROSPERO study protocol. In summary, we reasoned that the changes in the updated COSMIN methodology would not change the quality of the analysis. Moreover, breaching the study protocol would have decreased our trustworthiness in performing the data synthesis.

Another strength is the clearly defined construct that formed the basis for the selection of articles, ie, “capacity” in the ICF activity domain.^3^ This approach generated more homogeneous test content, which can make the results of the systematic review easier to interpret. Capacity denotes a patient's ability to perform an activity in a standardized environment and is useful to assess and compare patients’ functioning in a standardized way.^3^ However, it is important to note that results from a physical capacity task are not directly transferable to a patient's environment outside the test conditions, but rather signify the patient's *potential* of performing the activity outside the test conditions.

This review also has a few limitations. First, the COSMIN 4-point checklist was originally developed for PROMs,^32^ which could compromise its validity for assessing physical capacity tasks. However, we argue that the checklist is indeed appropriate for physical capacity tasks because the items of the checklist are phrased in a general manner and draw upon the researchers’ knowledge of the test at hand, such as the appropriate time interval between the first and second administrations in a reliability study. The checklist has also been successfully used in previous systematic reviews of physical capacity tasks for other patient groups.^33,35^

Another possible limitation is the exclusion of so-called functional capacity evaluations because we could not obtain the original test manuals for these tests due to copyright issues. Functional capacity evaluations are test batteries commonly used for return-to-work evaluations and could potentially have added value to this systematic review. However, we could not reliably determine the quality of the tests because we could not review the detailed procedures. Also, many of the excluded functional capacity evaluation articles appeared to focus on either the composite scores of the batteries or constructs such as “level of effort,” “work capacity,” and “safe lifting,” which would have led to the exclusion of several articles.

A final possible limitation is that the data extraction was not performed completely independently, as 1 author (M.J.) extracted the data and a second author (A.G., M.L., or R.S.) double-checked it for accuracy. In contrast, at least 2 authors independently performed all other methodological steps of the review. We decided on the current data extraction procedure because of the high accuracy of the first author in the pilot of this methodological step. We therefore argue that having a second author double-checking all data extracted was almost equivalent to having 2 authors independently performing the data extraction.

## Conclusion

In conclusion, the 5-repetition sit-to-stand, 5-minute walk, 50-ft walk, Progressive Isoinertial Lifting Evaluation, Timed “Up & Go,” and 1-minute stair-climbing tasks are promising physical capacity tasks for the measurement of functioning in patients with chronic LBP. However, more research on the measurement error and responsiveness of these tasks is needed to be able to fully recommend them as outcome measures in research and clinical practice for patients with LBP.

## Supplementary Material

pzy159_Supplemental_eAppendixs

## Clinical Trial or Systematic Review Registration

The study protocol was registered at the International Prospective Register of Systematic Reviews (PROSPERO; http://www.crd.york.ac.uk/PROSPERO; registration number: CRD42016042011).
