# Supplementary material for: Level of Evidence for Reliability, Validity, and Responsiveness of Physical Capacity Tasks Designed to Assess Functioning in Patients With Low Back Pain: A Systematic Review Using the COSMIN Standards
Source: Phys Ther. 2018 Dec 18;99(4):457–77. doi: 10.1093/ptj/pzy159 (PMC6488491; doi:10.1093/ptj/pzy159)
Supplement: pzy159_Supplemental_eAppendixs [file pzy159_supplemental_eappendixs.docx]

**APPENDIXES**

**Appendix 1. Complete search strategy, exemplified for Medline Ovid.**

| Field labels:   - .ti,ab.= titel & abstract - .kf. = keywords - / = MeSH - exp/ = MeSH, exploded - adjx = adjacent within x words |
| --- |
| 1. exp Back Pain/  2. Sciatica/  3. ((back or spinal or spine or lumbopelvic or lowback or lumbal or lumbar or lumbosacral or sciatic) adj2 (pain or ache* or neuralgia*)).ti,ab,kf.  4. ((lumbal or lumbar or lumbosacral) adj2 syndrome*).ti,ab,kf.  5. (backache or backpain or lumbago or sciatica or back disorder* or ischialgia).ti,ab,kf.  6. or/1-5  7. exp Severity of Illness Index/  8. exp Disability Evaluation/  9. ((physical or functional or lift* or walk) adj2 (task* or test* or assessment* or outcome* or evaluation* or capacity or performance)).ti,ab,kf.  10. ((capacity or performance) adj2 (test* or task* or measure* or outcome*)).ti,ab,kf.  11. test package*.ti,ab,kf.  12. (5-minute walk* or five-minute walk* or 5min-walk* or 6-minute walk* or six-minute walk* or 6min-walk* or 50-foot walk* or 50-ft walk* or sit-to-stand or chair stand* or timed up-and-go or stair climb* or forward reach or progressive isoinertial lifting evaluation or shuttle walk test or isernhagen work systems functional capacity evaluation or work-well systems functional capacity evaluation or self-paced walking test or self-selected walking speed or sock test or pick-up test or lift test or roll-up test or villiger test or dynamic pull or stand-to-floor or rapid gait or back performance scale or shuttle walking test* or stand-to-floor or gesture evaluation test*).ti,ab,kf.  13. or/7-12  14. Instrumentation.fs.  15. Methods.fs.  16. Validation Studies.pt.  17. Comparative Study.pt.  18. Psychometrics/  19. exp "Outcome Assessment (Health Care)"/  20. Observer Variation/  21. exp Health Status Indicators/  22. exp Reproducibility of Results/  23. Discriminant Analysis/  24. (psychometr* or outcome assessment or observer variation or reproducib* or reliab* or unreliab* or valid* or coefficient or homogeneity or homogeneous or "internal consistency" or (cronbach* and (alpha or alphas)) or (item and (correlation* or selection* or reduction*)) or agreement or precision or imprecision or "precise values" or test-retest or (test and retest) or (reliab* and (test or retest)) or stability or interrater or inter-rater or intrarater or intra-rater or intertester or inter-tester or intratester or intra-tester or interobserver or inter-observer or intraobserver or intraobserver or intertechnician or inter-technician or intratechnician or intra-technician or interexaminer or inter-examiner or intraexaminer or intra-examiner or interassay or inter-assay or intraassay or intra-assay or interindividual or inter-individual or intraindividual or intra-individual or interparticipant or inter-participant or intraparticipant or intra-participant or kappa or kappa's or kappas or repeatab* or ((replicab* or repeated) and (measure or measures or findings or result or results or test or tests)) or generaliza* or generalisa* or concordance or (intraclass and correlation*) or discriminative or "known group" or factor analysis or factor analyses or dimension* or subscale* or (multitrait and scaling and (analysis or analyses)) or item discriminant or interscale correlation* or error or errors or "individual variability" or (variability and (analysis or values)) or (uncertainty and (measurement or measuring)) or "standard error of measurement" or sensitiv* or responsive* or (((minimal or minimally or clinical or clinically) and (important or significant or detectable) and change) or difference) or (small* and (real or detectable) and (change or difference)) or meaningful change or "ceiling effect" or "floor effect" or "Item response model" or IRT or Rasch or "Differential item functioning" or DIF or "computer adaptive testing" or "item bank" or "cross-cultural equivalence").ti,ab,kf.  25. (clinimetr* or clinometr* or outcome measure*).mp.  26. or/14-25  27. 6 and 13 and 26  28. (animals not humans).sh.  29. 27 not 28  30. remove duplicates from 29  31. (addresses or biography or case reports or comment or directory or editorial or festschrift or interview or lectures or legal cases or legislation or letter or news or newspaper article or patient education handout or popular works or congresses or consensus development conference or consensus development conference, nih or practice guideline).pt.  32. 30 not 31 |

**Appendix 2. Derivation of validity hypotheses used in the data synthesis.**

| Article | Methods used for investigating validity | Phrases in the articles that suggest what was expected in relation to validity | Hypotheses used in the data synthesis |
| --- | --- | --- | --- |
| Conway, 2011^1^ | “Pearson product moment correlation coefficients were used to examine the strength of relationships between variables.” | "We hypothesized that self-reported walking disability would be more highly correlated with performance than with capacity." | Convergent validity: Self-reported walking disability [Quebec Back Pain Disability Questionnaire, Oswestry Disability Index, Pain Disability Index, the physical function subscale of the 36-item short form survey and the Symptom Severity subscales of the Swiss Spinal Stenosis Questionnaire], will be more highly correlated with performance [physical activity monitor] than with capacity [the self-paced walking test]. |
| Gautschi, 2016a^2^ | “The relationship between TUG test times and various subjective outcome measures was measured using Pearson’s correlation coefficient.” | “In every case, the correlation was in the correct direction (e.g., as the health-related quality of life decreased, the Timed Up and Go test times increased).” | Convergent validity: Timed up-and-go will be positively correlated with (i) visual analogue scale on back pain, (ii) the Roland-Morris Disability Index and (iii) the Oswestry Disability Index, and be inversely correlated with (iv) EuroQoL-5D index and (v) EuroQoL-5D visual analogue scale as well as (vi) the physical component summary and (vii) the mental component summary of the 12-item short form survey. |
| Kahraman, 2016^3^ | “Concurrent validity was determined by calculating Spearman’s rank correlation coefficients between the Oswestry Disability Index and the visual analogue scale.” | “The 30-s chair stand test was significantly correlated with Oswestry Disability Index and pain intensity at activity. The 30-s chair stand test can reflect a participant’s disability and pain related to LBP. These findings suggest that the 30-s chair stand test has adequate validity for use in patients with nonspecific LBP.” | Convergent validity: The 30-s chair stand will show statistically significant correlations with Oswestry Disability Index and pain intensity during activity. |
| Lee, 2001^4^ | “Pearson’s correlation coefficient (r) statistics were used for the correlations between the total Roland-Morris Disability Questionnnaire (RMDQ) scores and performance tests. Selected RMDQ items were analyzed separately across subjects and correlated with the PPT specific tests (table 1) by using point-biserial correlation (rpb) statistics.” | “The moderate correlations between total RMDQ scores and PPT were expected because clinician-measured physical function (PPT) is one of the dimensions measured by self-reporting activity limitation (RMDQ). “  [Lee et al. did not provide thresholds for “moderate” correlations. Hence, we interpreted moderate correlations as r=0.30-050 according to the literature.^5^] | Convergent validity: Physical capacity tasks will correspond moderately (r=0.30-0.50) with the corresponding items in the Roland-Morris Disability Questionnaire. |
| Ocarino, 2009^6^ | “A Pearson product-moment analysis was used to investigate the relationship between the final score obtained in the Roland Morris questionnaire and the times obtained in the execution of the physical capacity tests: 50-foot walk test and sit-to-stand.” | [Unclear what was expected in relation to validity.] | – |
| Odebiyi, 2007^7^ | “Pearson product moment and Spearman’s rho correlation were used to determine significant relationships between variables.” | [Unclear what was expected in relation to validity.] | – |
| Rainville, 2012^8^ | “Pearson product moment correlations were used to compare the results from motorized treadmill test and self-paced walking test with self-reported walking measures (Oswestry Disability Index walking item score and the  Spinal Stenosis Questionnaire physical function subscale).” | “Results from both motorized treadmill test and self-paced walking test showed strong correlations with each other and self-reported walking abilities, offering further validation that both walking tests assess limitations that are characteristic of neurogenic claudication to a similar degree.”  [Rainville et al. did not provide thresholds for “strong” correlations. Hence, we interpreted strong correlations as r>0.50 according to the literature.^5^] | Convergent validity: The motorized treadmill test and self-paced walking test will show strong correlations (r>0.50) with each other and with the Oswestry Disability Index walking item score, the Spinal Stenosis Questionnaire physical function subscale, self-estimated walking time and self-estimated distance. |
| Simmonds, 1998^9^ | “Pearson’s correlation coefficients were calculated and the correlation matrices were examined for evidence of convergent and discriminant validity among pain, disability, and physical performance measures within groups.“  “A MANOVA was used to determine whether there were differences in physical performance between groups.“ | 1. "Evidence of convergent and discriminative validity was supported through stronger correlational patterns among the performance measures and weaker correlations between performance and external measures"  2. “It was anticipated that tasks that comprised similar performance characteristics would correlate most strongly. For example, all tasks that involved walking were highly correlative with each other. (...) In contrast, it was anticipated that tasks that comprised dissimilar performance characteristics would correlate weakly.”  3. ”Performance differences between LBP and control groups on most of the tasks (...) supports the face validity of most of the tasks”.  [Simmonds et al. used the term *face* *validity*, but we interpret it as known group validity according to the COSMIN taxonomy.^10^] | 1. Convergent validity: There will be a stronger correlation between the 5-minute walking, 50-foot walk (fast and preferred speed), timed up-and-go and 5-repetition sit-to-stand compared to their correlations with (i) pain intensity, (ii) pain affect and the (iii) Roland-Morris disability scale.  2. Convergent validity: 5-minute walking, 50-foot walk (fast and preferred speed) and timed up-and-go will correlate more strongly with each other compared to their correlations with (iv) lumbar flexion, (v) repeated trunk flexion, (vi) 5-repetition sit-to-stand, (vii) loaded reach, (viii) unloaded reach and Sorensen test.  3. Known group validity: Patients with LBP will have worse results on the physical capacity tasks compared to the control group. [We regard this hypothesis as known group validity accor^10^ although Simmonds et al. refer to it as face validity.] |
| Soer, 2006^11^ | “To answer the question whether both tests measure the same outcome, descriptives, Pearson correlation coefficients, 95% confidence intervals of the mean difference and dependent t tests were calculated.” | “The criteria for good comparison were set as for concurrent validity: Pearson correlation coefficient higher than 0.75 (...) for the differences of the progressive isoinertial lifting evaluation and the Work-Well Systems Functional Capacity Evaluation lifting test.” | Convergent validity: Progressive Isoinertial Lifting Evaluation and the Work-Well Systems Functional Capacity Evaluation lifting test will correlate >0.75. |
| Staartjes, 2018^12^ | **“Pearson correlation was used to assess the correlation between log_10_-transformed 5R-STS [5-repetition sit-to-stand] test times and validated questionnaires.”** | **“We observed a direct correlation of logarithmically transformed 5R-STS test times and functional impairment (Fig. 2), as measured by RMDQ (r = 0.49, 95% CI 0.36– 0.60) and ODI (r = 0.44, 95% CI 0.30–0.56), as well as with VAS back pain severity (r = 0.31, 95% CI 0.16–0.45) (all p < 0.001). There was no relevant correlation with VAS leg pain severity (p = 0.207, Fig. 3). The 5R-STS test also demonstrated indirect correlation with HRQOL (Fig. 4), as measured by EQ-5D index (r = -0.41, 95% CI -0.53 to 0.27, p < 0.001) but not with EQ-5D VAS score (p = 0.091).”** | **Convergent validity: Log_10_-transformed 5-repetition sit-to-stand will show statistically significant correlations with (i) Roland-Morris Disability Questionnaire, (ii) Oswestry Disability Index, (iii) visual analogue scale on back pain severity, (iv) visual analogue scale on leg severity, (v) EuroQoL-5D index, and (vi) EuroQoL-5D visual analogue scale.** |
| Strand, 2011^13^ | “A priori hypotheses of construct validity and responsiveness were defined regarding the expected associations between the physical tests and the self-report measures.” | [Clear a priori hypotheses are presented in the article.] | Convergent validity: The scores of physical tests of activities [PILE, lift test and 15-meter walk] were expected to be moderately correlated (.60>r≥.30) with scores of self-report questionnaires of functioning [the Hannover Functional Ability Questionnaire than with scores of the Roland-Morris Disability Questionnaire].  Convergent validity: Scores of all physical tests were expected to be more highly correlated with scores of the Hannover Functional Ability Questionnaire than with scores of the Roland-Morris Disability Questionnaire. |
| Teixeira, 2010^14^ | “The correlation between the functional tests and the questionnaires used Spearman correlation.”  “Group comparisons were conducted by using paired t-tests or the nonparametric equivalent when a non-normal distribution was observed or when the data were ordinal.” | 1. “The physical performance test scores did not correlate strongly with the level of pain as measured by a VAS, but they correlated moderately with self-perception of the level of limitation (RMDQ) and with each other, supporting the validity of these tests.”  2. [There seems to be no specific phrase that explicitly outlines what was expected in relation to known group validity. However, the discussion suggests that it was expected that patients with lumbar spinal stenosis would have statistically significant worse scores compared to apparently healthy persons.] | Convergent validity: There will be stronger correlations among the included tests [50-foot walk, 5-minute walk, sit stand, timed up and go] and with Roland-Morris Disability Questionnaire compared to correlations between the included tests and pain intensity.  Known group validity: Patients with low back pain will have worse scores on the included tests compared to persons with no complaints of LBP. |
| Whitehurst, 2001^15^ | “An analysis of variance between groups were performed. “ | [There seems to be no specific phrase that explicitly outlines what was expected in relation to validity. However, the discussion suggests that it was expected that patients with lumbar spinal stenosis would have worse scores compared to apparently healthy persons.] | Known group validity: Patients with lumbar spinal stenosis will have worse results on the included tests compared to apparently healthy persons. |

**References for Appendix 2^9,13,14^**

**^,16-19^**

1. Conway J, Tomkins CC, Haig AJ. Walking assessment in people with lumbar spinal stenosis: capacity, performance, and self-report measures. *Spine J.* 2011;11(9):816-823.

2. Gautschi OP, Smoll NR, Corniola MV, et al. Validity and Reliability of a Measurement of Objective Functional Impairment in Lumbar Degenerative Disc Disease: The Timed Up and Go (TUG) Test. *Neurosurgery.* 2016;79(2):270-278.

3. Kahraman T, Ozcan Kahraman B, Salik Sengul Y, Kalemci O. Assessment of sit-to-stand movement in nonspecific low back pain: a comparison study for psychometric properties of field-based and laboratory-based methods. *Int J Rehabil Res.* 2016;39(2):165-170.

4. Lee CE, Simmonds MJ, Novy DM, Jones S. Self-reports and clinician-measured physical function among patients with low back pain: a comparison. *Arch Phys Med Rehabil.* 2001;82(2):227-231.

5. Prinsen CAC, Mokkink LB, Bouter LM, et al. COSMIN guideline for systematic reviews of patient-reported outcome measures. *Qual Life Res.* 2018;27(5):1147-1157.

6. Ocarino J, Gonçalves G, Vaz D, Cabral A, Porto J, Silva M. Correlation between a functional performance questionnaire and physical performance tests among patients with low back pain. *Brazilian Journal of Physical Therapy.* 2009;13(4):343-349.

7. Odebiyi DO, Kujero SO, Lawal TA. Relationship Between Spinal Mobility, Physical Performance, Pain Intensity and Functional Disability In Patients With Chronic Low Back Pain. *Nigerian Journal of Medical Rehabilitation.* 2007;11(2):49-54.

8. Rainville J, Childs LA, Pena EB, et al. Quantification of walking ability in subjects with neurogenic claudication from lumbar spinal stenosis--a comparative study. *Spine J.* 2012;12(2):101-109.

9. Simmonds MJ, Olson SL, Jones S, et al. Psychometric characteristics and clinical usefulness of physical performance tests in patients with low back pain. *Spine.* 1998;23(22):2412-2421.

10. Mokkink LB, Terwee CB, Patrick DL, et al. The COSMIN study reached international consensus on taxonomy, terminology, and definitions of measurement properties for health-related patient-reported outcomes. *J Clin Epidemiol.* 2010;63(7):737-745.

11. Soer R, Poels BJ, Geertzen JH, Reneman MF. A comparison of two lifting assessment approaches in patients with chronic low back pain. *J Occup Rehabil.* 2006;16(4):639-646.

12. Staartjes VE, Schroder ML. The five-repetition sit-to-stand test: evaluation of a simple and objective tool for the assessment of degenerative pathologies of the lumbar spine. *J Neurosurg Spine.* 2018:1-8.

13. Strand LI, Anderson B, Lygren H, Skouen JS, Ostelo R, Magnussen LH. Responsiveness to change of 10 physical tests used for patients with back pain. *Phys Ther.* 2011;91(3):404-415.

14. Teixeira Da Cunha-Filho I, Lima FC, Guimarães FR, Leite HR. Use of physical performance tests in a group of Brazilian Portuguese-speaking individuals with low back pain. *Physiotherapy Theory and Practice.* 2010;26(1):49-55.

15. Whitehurst M, Brown LE, Eidelson SG, D'Angelo A. Functional mobility performance in an elderly population with lumbar spinal stenosis. *Arch Phys Med Rehabil.* 2001;82(4):464-467.

16. Moradi B, Benedetti J, Zahlten-Hinguranage A, Schiltenwolf M, Neubauer E. The value of physical performance tests for predicting therapy outcome in patients with subacute low back pain: A prospective cohort study. *European Spine Journal.* 2009;18(7):1041-1049.

17. Strand LI, Moe-Nilssen R, Ljunggren AE. Back Performance Scale for the assessment of mobility-related activities in people with back pain. *Phys Ther.* 2002;82(12):1213-1223.

18. Terwee CB, Bot SDM, de Boer MR, et al. Quality criteria were proposed for measurement properties of health status questionnaires. *Journal of Clinical Epidemiology.* 2007;60(1):34-42.

19. Terwee C, Mokkink L, Knol D, Ostelo RJG, Bouter L, de Vet HW. Rating the methodological quality in systematic reviews of studies on measurement properties: a scoring system for the COSMIN checklist. *Quality of Life Research.* 2012;21(4):651-657.

**Appendix 3. Derivation of responsiveness hypotheses used in the data synthesis.**

| Article | Methods used for investigating responsiveness | Phrases in the articles that suggest what was expected in relation to responsiveness | Derived hypotheses used in the data synthesis |
| --- | --- | --- | --- |
| Andersson, 2010^51^ | “Responsiveness of the performance tests was measured by calculating the area under the receiver operating characteristic (ROC) curve (AUC), using the data of participants who stayed unchanged or improved.” | “We considered an AUC of 0.70 or above to indicate that a test has adequate responsiveness.” | The physical capacity tasks will be able to discriminate between patients who stayed unchanged or improved on a global perceived effect scale “LBP associated disability” (area under the receiver operating characteristics curve ≥0.70). |
| Campbell, 2006^38^ | Calculations of effect sizes, standardized response means, modified response means and area under the receiver operating characteristics curve. | [For calculations of area under the receiver operating characteristics curve, no threshold for sufficient responsiveness was given by Cambell et al. Hence, we used the generally accepted threshold of ≥0.70 for the hypothesis that we used in the data synthesis.^66^]  Measures of responsiveness not included in the data synthesis:  [We could not find what was expected for the results of effect sizes, standardized response means and modified response means in relation to responsiveness. Hence, we generated no hypotheses for these measures.] | The Shuttle walking test will be able to discriminate between patients who perceive their health as having improved between baseline and 12 months and those who consider their health to have remained stable (area under the receiver operating characteristics curve ≥0.70), |
| Gautschi, 2016b^45^ | “The relationship between TUG [timed up-and go] test times and PROMs before, as well as at W6 [6 weeks] postoperatively, was measured using Pearson correlation coefficient.” | “Validity is found to be present if the direction of the TUG t-scores changes correlates with the PROMs [patient-reported outcome measures].”  [Gautschi et al. used the term *validity*, but as the analyses concerns change scores, we interpret it as responsiveness according to the COSMIN taxonomy.^29^] | The change scores timed up-and-go t-scores will be positively correlated with (i) visual analogue scale on back pain, (ii) the Roland-Morris Disability Index and the (iii) Oswestry Disability Index, and be negatively correlated with (iv) EuroQoL-5D index, (v) the physical component summary and (vi) the mental component summary of the 12-item short form survey. |
| Rainville, 2012^41^ | 1. “Receiver operating characteristic (ROC) curves were plotted, and area under the curve (AUC) was calculated to explore the degree to which specific values of change scores for self-paced walking test and motorized treadmill test walking times and distances correctly reflect the reference standard of improved or not improved clinical status.”  2. “This study used two statistical methods to evaluate internal responsiveness. First, paired-sample t tests were calculated to assess the changes between initial and final self-paced walking test and motorized treadmill test scores. Next, standardized effect size (ES) was used to assess the magnitude of changes in self-paced walking test and motorized treadmill test compared with the variance (standard deviation [SD]) in those measures at baseline assessment.” | 1. [For calculations of area under the receiver operating characteristics curve, no threshold for sufficient responsiveness was given by the Rainville et al. Hence, we used the generally accepted threshold of ≥0.70 for the hypothesis that we used in the data synthesis.^66^  2. “(...) it was our assumption that actual limitations in walking ability were the major concern for these patients, and this would be reflected in strong relationships between changes in walking abilities and other outcomes’ dimensions.”  [Rainville et al. did not provide thresholds for “strong” correlations. Hence, we interpreted strong correlations as r>0.50 according to the literature.^66^]  Measures of responsiveness not included in the data synthesis:  [We did not pose any hypothesis for paired-sample t-tests since this measure of responsiveness has been recommended against The P-value from paired t-tests have been argued to be a measure of the statistical significance of the change scores instead of the validity of the change scores.^23^ Hence, we generated no hypotheses for t-tests.]  [For effect sizes, Rainville et al. provided values that indicate small- moderate- and large-magnitude responsiveness. However, this method for determining responsiveness has been recommended against since effect sizes measure the magnitude of the change scores, rather than the validity of the change scores.^23^ Hence, we did not generate any hypothesis for calculations of effect sizes. ] | 1. The motorized treadmill test and the self-paced walking test will be able to discriminate between improved and not improved patients regarding symptoms with walking after treatment (area under the receiver operating characteristics curve ≥0.70),  2. The change scores of the motorized treadmill test and the self-paced walking test will correlate strongly (r>0.50 ) with the Oswestry Disability Index walking item score, the Spinal Stenosis Questionnaire physical function subscale, self-estimated walking time and self-estimated distance. |
| Taylor, 2001^49^ | “Standardized effect sizes were calculated by use of the formula: (mean post-treatment score – Effect size = mean pre-treatment score) Standard deviation of the change scores Cohen (1977) defined an effect size of less than 0.2 as small, one of 0.5 as moderate and one of greater than 0.8 as large, the higher the effect size the greater the ability of a test instrument to detect clinically important change. An effect size of 1.0 is the equivalent of one SD in the sample.” | “If it [the shuttle walking test] is used to assess treatment that does not aim to increase physical function its responsiveness is likely to be reduced. This can be observed when comparing the effect size for the patients in the randomized controlled trial fitness group. The patients who underwent fitness training achieved an effect size of 1.42, against that of the patients within the randomized controlled trial control group which only reached 0.23. The fitness group also showed a greater improvement after treatment than that of the patients in the responsiveness orthopaedic clinic (ROC) group (effect size 0.94) as demonstrated by the larger effect size.” | The shuttle walking test will show larger effect sizes for a treatment aimed at increasing physical function compared to a treatment aimed at relieving symptoms. |
| Strand, 2002^56^ | Responsiveness of the back performance scale was examined by receiver operating characteristics curve statistics. | [For calculations of area under the receiver operating characteristics curve, no threshold for sufficient responsiveness was given by Strand et al. Hence, we used the generally accepted threshold of ≥0.70 for the hypothesis that we used in the data synthesis.^66^]  [The responsiveness hypothesis regarding effect sizes were not included since it only concerns the composite score of the back performance scale.] | The lift test will be able to discriminate between patients who have returned to work or not, one year after treatment (area under the receiver operating characteristics curve ≥0.70). |
| Strand, 2011^48^ | Methods: “A priori hypotheses of construct validity and responsiveness were defined regarding the expected associations between the physical tests and the self-report measures.” | [Clear a priori hypotheses are presented in the article.] | 1. Change scores of the physical tests of activities were expected to be moderately correlated with change scores of the self-report questionnaires of functioning.  2. Change scores of the physical tests were expected to be more highly correlated with change scores of the Hannover Functional Ability Questionnaire than with change scores of the Roland-Morris Disability Questionnaire.  3. Change scores of all physical tests were expected to show decreasing values according to decreasing improvement as reported on the Patient Global Impression of Change.  4. Change scores of all physical tests were expected to distinguish between patients with and without meaningful improvement as reported on Patient Global Impression of Change (very much, much, or slightly improved versus no change).  5. Change scores of all physical tests were expected to distinguish between patients with and without meaningful improvement in self-reported ability to work after rehabilitation.  6. The physical tests were expected to distinguish clinically important change from measurement error. |

**References for Appendix 3**

1. Andersson EI, Lin CC, Smeets RJ. Performance tests in people with chronic low back pain: responsiveness and minimal clinically important change. *Spine (Phila Pa 1976).* 2010;35(26):E1559-1563.

2. Campbell H, Rivero-Arias O, Johnston K, Gray A, Fairbank J, Frost H. Responsiveness of objective, disease-specific, and generic outcome measures in patients with chronic low back pain: an assessment for improving, stable, and deteriorating patients. *Spine (Phila Pa 1976).* 2006;31(7):815-822.

3. Prinsen CAC, Mokkink LB, Bouter LM, et al. COSMIN guideline for systematic reviews of patient-reported outcome measures. *Qual Life Res.* 2018;27(5):1147-1157.

4. Gautschi OP, Joswig H, Corniola MV, et al. Pre- and postoperative correlation of patient-reported outcome measures with standardized Timed Up and Go (TUG) test results in lumbar degenerative disc disease. *Acta Neurochir (Wien).* 2016;158(10):1875-1881.

5. Mokkink LB, Terwee CB, Patrick DL, et al. The COSMIN study reached international consensus on taxonomy, terminology, and definitions of measurement properties for health-related patient-reported outcomes. *J Clin Epidemiol.* 2010;63(7):737-745.

6. Rainville J, Childs LA, Pena EB, et al. Quantification of walking ability in subjects with neurogenic claudication from lumbar spinal stenosis--a comparative study. *Spine J.* 2012;12(2):101-109.

7. de Vet HCW, Terwee CB, Mokkink LB, Knol DL. *Measurement in medicine: a practical guide.* Cambridge: Cambridge University Press; 2011.

8. Taylor S, Frost H, Taylor A, Barker K. Reliability and responsiveness of the shuttle walking test in patients with chronic low back pain. *Physiother Res Int.* 2001;6(3):170-178.

9. Strand LI, Moe-Nilssen R, Ljunggren AE. Back Performance Scale for the assessment of mobility-related activities in people with back pain. *Phys Ther.* 2002;82(12):1213-1223.

10. Strand LI, Anderson B, Lygren H, Skouen JS, Ostelo R, Magnussen LH. Responsiveness to change of 10 physical tests used for patients with back pain. *Phys Ther.* 2011;91(3):404-415.

eTable 1. COSMIN methodological quality ratings, result ratings and level of evidence for construct validity and criterion validity presented per physical capacity task.

| **Physical capacity task** | **Article** | **Validity design** | **Sample size** | **COSMIN**  **score^32^** | **Result rating (+/?/-)^30^** | **Best evidence synthesis:  Level of evidence** |
| --- | --- | --- | --- | --- | --- | --- |
| **30-second chair stand test** | Kahraman, 2016 ^47^ | Hypothesis testing (convergent validity) | 38 | Good* | Convergent validity: 1/1 hypotheses confirmed (+) | **Hypothesis testing:** Limited (+) |
| **5-repetiton sit-to-stand** | Lee, 2001^19^ | Hypothesis testing (convergent validity) | 83 | Fair | 1/1 hypothesis confirmed (+) | **Hypothesis testing:** Moderate (+) |
|  | Ocarino, 2009^53^ | Not clear | 30 | Poor** | No hypotheses posed (?) |  |
|  | Odebiyi, 2007^54^ | Not clear | 23 | Poor** | No hypotheses posed (?) |  |
|  | Simmonds, 1998^17^ | Hypothesis testing (convergent validity and known group validity) | 44 | Fair | Convergent validity:  7/7 hypotheses confirmed (+) Known group validity:  1/1 hypothesis confirmed (+) |  |
|  | **Staartjes, 2018^46^** | **Hypothesis testing (convergent validity)** |  | **Fair** | **4/6 hypotheses confirmed (-)** |  |
|  | Teixeira da Cunha-Filho, 2010^50^ | Hypothesis testing (convergent validity and known group validity) | 30 | Fair | Convergent validity:  1/1 hypothesis confirmed (+)  Known group validity:  1/1 hypothesis confirmed (+) |  |
| **50-foot walk** | Lee, 2001^19^ | Hypothesis testing (convergent validity) | 83 | Fair | Convergent validity:  1/1 hypothesis confirmed (+) | **Hypothesis testing:** Moderate (+) |
|  | Ocarino, 2009^53^ | Not clear | 30 | Poor** | No hypotheses posed (?) |  |
|  | Odebiyi, 2007^54^ | Hypothesis testing (convergent validity) | 23 | Poor** | No hypotheses posed (?) |  |
|  | Simmonds, 1998^17^ | Hypothesis testing (convergent validity and known group validity) | 44 | Fair | Convergent validity: 8/8 hypotheses confirmed (+)  Known group validity: 1/1 hypothesis confirmed (+) |  |
|  | Strand, 2011^48^ | Hypothesis testing (convergent validity) | 98 | Good | Convergent validity:  2/2 hypotheses confirmed (+) |  |
|  | Teixeira da Cunha-Filho, 2010^50^ | Hypothesis testing (convergent validity and known group validity) | 30 | Fair | Convergent validity:  1/1 hypothesis confirmed (+) Known group validity:  1/1 hypothesis confirmed (+) |  |
| **50-foot walk, preferred speed** | Simmonds, 1998^17^ | Hypothesis testing (convergent validity an known group validity) | 44 | Fair | Convergent validity:  8/8 hypotheses confirmed Known group validity: 0/1 hypothesis confirmed (-) | **Hypothesis testing:** Conflicting |
| **5-minute walk** | Lee, 2001^19^ | Hypothesis testing (convergent validity) | 83 | Fair | Convergent validity:  1/1 hypothesis confirmed (+) | **Hypothesis testing:** Moderate (+) |
|  | Odebiyi, 2007^54^ | Not clear | 23 | Poor** | No hypotheses posed (?) |  |
|  | Simmonds, 1998^17^ | Hypothesis testing (convergent validity and known group validity) | 44 | Fair | Convergent validity:  8/8 hypotheses confirmed (+) Known group validity:  1/1 hypothesis confirmed (+) |  |
|  | Teixeira da Cunha-Filho, 2010^50^ | Hypothesis testing (convergent validity and known group validity) | 30 | Fair | Convergent validity:  1/1 hypothesis confirmed (+) Known group validity:  1/1 hypothesis confirmed (+) |  |
| **Lift test, modified** | Strand, 2011^48^ | Hypothesis testing (convergent validity) | 98 | Good | Convergent validity:  2/2 hypotheses confirmed (+) | **Hypothesis testing:** Moderate (+) |
| **Motorized treadmill test** | Rainville, 2012^41^ | Hypothesis testing (convergent validity) | 50 | Fair | Convergent validity:  1/1 hypothesis confirmed (+) | **Hypothesis testing, LSS:** Limited (+) |
| **Progressive isoinertial lifting evaluation** | Strand, 2011^48^ | Hypothesis testing (convergent validity) | 98 | Good | Convergent validity: 2/2 hypotheses confirmed (+) | **Hypothesis testing:** Moderate (+) |
|  | Soer, 2006^55^ | Hypothesis testing (convergent validity) | 53 | Fair | Convergent validity: 1/1 hypothesis confirmed (+) |  |
| **Self-paced walking test** | Conway, 2011^20^ | Hypothesis testing (convergent validity) | 12 | Fair | Convergent validity:  0/1 hypothesis confirmed (-) | **Hypothesis testing, LSS:** Conflicting |
|  | Rainville, 2012^41^ | Hypothesis testing (convergent validity) | 50 | Fair | Convergent validity:  1/1 hypothesis confirmed (+) |  |
| **Timed up-and-go** | Simmonds, 1998^17^ | Hypothesis testing (convergent validity and known group validity) | 44 | Fair | Convergent validity: 8/8 hypotheses confirmed (+) Known group validity: 1/1 hypothesis confirmed (+) | **Hypothesis testing:** Moderate (+)  **Hypothesis testing, LDH/LSS/DDD:** Limited (+) |
|  | Teixeira da Cunha-Filho, 2010^50^ | Hypothesis testing (convergent validity and known group validity) | 30 | Fair | Convergent validity:  1/1 hypothesis confirmed (+) Known group validity: 1/1 hypothesis confirmed (+) |  |
|  | Gautschi, 2016a^15^ | Hypothesis testing (convergent validity) | 253 | Fair | Convergent validity:  7/7 hypotheses confirmed (+) |  |
| **Treadmill protocol** | Tomkins, 2009^42^ | Criterion validity | 45 | Good | Correlation with gold standard: 0.88 (+) | **Criterion validity, LSS:** Limited (+) |
| **Treadmill walking test** | Whitehurst, 2001^43^ | Hypothesis testing (known group validity) | 57 | Fair | Known group validity: 1/1 hypothesis confirmed (+) | **Hypothesis testing, LSS:** Limited (+) |
| **Weight carrying test** | Whitehurst, 2001^43^ | Hypothesis testing (known group validity) | 57 | Fair | Known group validity:  1/1 hypothesis confirmed (+) | **Hypothesis testing, LSS:** Limited (+) |

+ = Positive rating: 75% of the results are in accordance with the hypotheses (hypothesis testing); correlation with gold standard ≥ 0.70 OR AUC ≥ 0.70 (criterion validity)
? = Indeterminate rating: No hypotheses defined (hypothesis testing); Not all information for ‘+’ reported (criterion validity)
- = Negative rating: Criteria for ‘+’ not met
* = Rating changed after removal of sample size item from “worst score counts” summary in the COSMIN 4-point checklist
** = Not included in data synthesis due to poor methodological quality score
AUC = area under the receiver operating characteristics curve; COSMIN = COnsensus-based Standards for the selection of health Measurement INstruments, DDD = lumbar degenerative disc disease, LDH = lumbar disc herniation, LSS = lumbar spinal stenosis

eTable 2. COSMIN methodological quality ratings, result ratings and level of evidence for responsiveness presented per physical capacity task.

| **Physical capacity tasks** | **Study** | **Responsiveness design** | **Sample size^a^** | **COSMIN**  **score^32^** | **Result rating (+/?/-)^30^** | **Best evidence synthesis:  Level of evidence** |
| --- | --- | --- | --- | --- | --- | --- |
| **1-minute stair-climbing** | Andersson, 2010^51^ | Construct approach | 178 | Fair | 1/1 hypothesis confirmed (+) | Limited (+) |
| **5-repetition sit-to-stand** | Andersson, 2010^51^ | Construct approach | 178 | Fair | 1/1 hypothesis confirmed (+) | Limited (+) |
| **50-foot walk** | Andersson, 2010^51^ | Construct approach | 177 | Fair | 0/1 hypothesis confirmed (-) | Moderate (-) |
|  | Strand, 2011^48^ | Construct approach | 98 | Good | 1/6 hypotheses confirmed (-) |  |
| **5-minute walk** | Andersson, 2010^51^ | Construct approach | 176 | Fair | 0/1 hypothesis confirmed (-) | Limited (-) |
| **Lift test** | Strand, 2002^56^ | Construct approach | 114 | Fair | 0/1 hypotheses confirmed (-) | Limited (-) |
| **Lift test, modified** | Strand, 2011^48^ | Construct approach | 98 | Good | 4/6 hypothesis confirmed (-) | Moderate (-) |
| **Motorized treadmill test** | Rainville, 2012^41^ | Construct approach | 50 | Fair | Walking time:  1/2 hypotheses confirmed (-)  Distance:  1/2 hypotheses confirmed (-) | LSS: Limited (-) |
| **Progressive isoinertial lifting evaluation** | Andersson, 2010^51^ | Construct approach | 167 | Fair | 0/1 hypothesis confirmed (-) | Moderate (-) |
|  | Strand, 2011^48^ | Construct approach | 98 | Good | 1/6 hypotheses confirmed (-) |  |
| **Self-paced walking test** | Rainville, 2012^41^ | Construct approach | 50 | Fair | Walking time: 0/2 hypotheses confirmed  Distance: 0/2 hypotheses confirmed | LSS: Limited (-) |
| **Shuttle walking test** | Campbell, 2006^38^ | Construct approach | 250 | Fair | 1/1 hypothesis confirmed (+) | Limited (+)  uLBP/PL/SP: Limited (+) |
|  | Taylor, 2001^49^ | Construct approach | 44 | Fair | 1/1 hypothesis confirmed (+) |  |
| **Timed up-and-go** | **Gautschi, 2016b^45^** | **Construct approach** | **136** | **Fair** | **6/6 hypotheses confirmed** | **LDH/LSS/DDD: Limited (+)** |

+ = Positive rating: 75% of the results are in accordance with the hypotheses OR AUC ≥ 0.70
? = Indeterminate rating: No hypotheses defined
- = Negative rating: Criteria for ‘+’ not met

AUC = area under the receiver operating characteristics curve, COSMIN = COnsensus-based Standards for the selection of health Measurement Instruments, LSS = lumbar spinal stenosis, PL = post-laminectomy, SP = spondylolisthesis, uLBP = unspecified LBP
